# Prevalence and associated factors of circadian rhythm sleep-wake disorders and insomnia among visually impaired Japanese individuals

**DOI:** 10.1186/s12889-020-09993-8

**Published:** 2021-01-06

**Authors:** Norihisa Tamura, Taeko Sasai-Sakuma, Yuko Morita, Masako Okawa, Shigeru Inoue, Yuichi Inoue

**Affiliations:** 1 Department of Psychology, Graduate School of Humanities and Social Sciences, Hiroshima University, Hiroshima, Japan; 2 Department of Somnology, Tokyo Medical University, Tokyo, Japan; 3 Japan Somnology Center, Neuropsychiatric Research Institute, Tokyo, Japan; 4 Department of Clinical Laboratory Science, Teikyo University, Tokyo, Japan; 5 Department of Liberal Arts, Faculty of Science and Technology, Tokyo University of Science, Chiba, Japan; 6 Yoyogi Sleep Disorder Center, Tokyo, Japan; 7 Japan Foundation for Neuroscience and Mental Health, Tokyo, Japan; 8 Department of Preventive Medicine and Public Health, Tokyo Medical University, Tokyo, Japan

**Keywords:** Circadian rhythm sleep-wake disorder, Light perception, Non-24-h sleep-wake rhythm type, Prevalence, Visual impairment

## Abstract

**Background:**

Although earlier studies have demonstrated that circadian rhythm sleep-wake disorders (CRSWD) are more prevalent in visually impaired individuals, the actual prevalence of CRSWD and insomnia among the visually impaired Japanese population remains unclear. The aim of this cross-sectional, telephone-based study was to estimate the prevalence of CRSWD and insomnia, and explore factors associated with CRSWD and insomnia among visually impaired Japanese individuals.

**Methods:**

A nationwide telephone survey was conducted among visually-impaired individuals through local branches of the Japan Federation of the Blind. In total, 157 visually impaired individuals were eligible for this study. Demographic information and information about visual impairments, lifestyle, and sleep patterns were assessed using questionnaires and subsequent telephone interviews. CRSWD and insomnia were defined according to the International Classification of Sleep Disorders-Third Edition criteria.

**Results:**

The prevalence of CRSWD in visually impaired individuals was 33.1%. Among those with CRSWD, a non-24-h/irregular sleep-wake rhythm type was the most frequently observed (26.8%), followed by an advanced sleep-wake phase type and a delayed sleep-wake phase type (3.8 and 2.5%, respectively). Furthermore, 28.7% of the visually impaired individuals were found to have insomnia. In the visually impaired individuals, the absence of light perception, unemployment, living alone, and use of hypnotics were significantly associated with CRSWD, whereas only the use of hypnotics was extracted as a marginally associated factor of insomnia.

**Conclusions:**

CRSWD and insomnia were highly prevalent in visually impaired Japanese individuals. The presence of CRSWD among the visually impaired individuals was associated with a lack of light perception and/or social zeitgebers.

## Background

Circadian rhythm sleep-wake disorders (CRSWD) and insomnia are frequently observed in visually impaired individuals (13–63% and 43–96%, respectively) [1–8]. It has also been reported that the prevalence of these disorders is higher in visually impaired individuals without light perception (LP) than in those with LP [5–11]. In visually impaired individuals without LP, circadian rhythm-related problems are associated with their lowered mental/physical quality of life [11]. Several epidemiological studies performed in the United States, Europe, and New Zealand have reported that the prevalence of CRSWD ranges from 18 to 39%, while that of insomnia ranges from 54 to 86% in visually impaired individuals without LP [2, 8, 9]. Consistent with these reports, sleep-related problems were frequently observed among visually impaired Japanese individuals in our preliminary survey who were identified through a single questionnaire [11]; however, no relevant studies assessing detailed information about the sleep patterns, including bedtime and wake-time, or total sleep time, of visually impaired individuals have been performed in Japan. Thus, the actual prevalence of CRSWD and insomnia among the visually impaired Japanese population remains unclear.

The occurrence of CRSWD and insomnia are intimately associated with visual loss or inhibited LP [6, 9, 10]. So far, no studies have described the factors associated with these sleep disorders other than clinical information of visual impairment, such as onset, cause, or status [12]. Meanwhile, it has been noted that the visually-impaired individuals without CRSWD are entrained to the 24-h social day via non-photic time cues, including strict scheduling of activities, exercise, mealtimes, and social interaction [10, 13–15]. Therefore, an absence of these non-photic zeitgebers may be associated with the development of CRSWD and insomnia.

The aims of the present study were to estimate the prevalence of CRSWD and insomnia, and explore factors associated with CRSWD and insomnia among visually impaired Japanese individuals.

## Methods

### Study setting and survey procedures

The study consisted of telephone surveys carried out between October 2014 and January 2015. The study was performed in cooperation with a non-profit organization, the Japan Federation of the Blind (JFB). The JFB is a nationwide organization consisting of 61 affiliated organizations that include approximately 50,000 blind or visually impaired members. The organization provides employment support, audio and Braille libraries, social and medical information, and various support services to visually impaired individuals in Japan.

Prior to the present study, the aforementioned preliminary questionnaire survey of sleep-related problems in visually impaired individuals was administered between October 2013 and November 2013 [11]. In the preliminary survey, we provided 61 affiliated organizations with study information through the JFB and asked for their cooperation. Of the 61 affiliated organizations, 60 agreed to cooperate in the preliminary survey. A total of 1200 visually impaired individuals were randomly selected according to a stratified random sampling method, in which the strata were formed based on gender and age. Among the 1200 eligible participants, 631 agreed to participate in the preliminary survey and completed the study questionnaires (response rate: 52.3%). Of the 631 responders, study information was only provided to the participants who agreed to participate in the secondary survey through the JFB (*n* = 500). The JFB distributed a letter in Braille and regular text asking individuals to cooperate in the secondary survey. The participants provided their responses and basic information (name, address, and phone number) and sent the information back to the Tokyo Medical University. Written informed consent was obtained from the participants and/or their family members.

In the secondary survey, telephone interviews were conducted by one of the authors (NT) and a staff member who had experience in conducting telephone surveys on large populations. The staff received 2 days of training prior to starting the survey administration. The survey consisted of a 30-min interview containing approximately 60 items relating to visual status and sleep habits. A detailed interview form is in an additional file [see Additional file 1]. Before conducting the main survey, the study staff also performed a pilot survey on 20 individuals in order to confirm the participants’ understanding of the questionnaire items, the results of which were excluded from the main survey. The study protocol, which adheres to the tenets of the Declaration of Helsinki, was reviewed and approved by the Research Ethics Committee of Tokyo Medical University (approval number: 2474).

### Participants

**Fig. 1:**
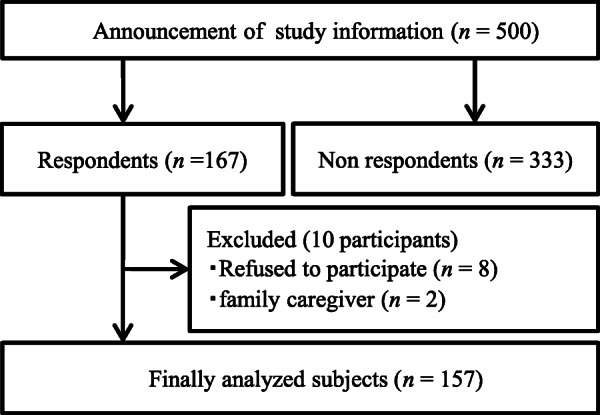
Subject flow diagram

Individuals with severe visual impairment who had Grade 1 (0.01 or less visual acuity in bilateral eyes) or Grade 2 ([1] monocular blindness and 0.02 or less vision in the other eye, or [2] 0.02 or less vision in bilateral eyes) visual disability were recruited. A total of 500 eligible participants were recruited, of whom, 167 returned responses. Of the 167 responders, eight participants who refused telephone interviews and data from two family caregivers were excluded. Consequently, data for 157 individuals (response rate: 31.4%) were analyzed (Fig. 1).

### Measurements

The questionnaire included the following information: age, gender (female/male), employment status (employed/unemployed; job category; number of working days per week; engagement in shift-work), residential status (living alone/living with family member), use of public assistance (yes/no; total time per week), body mass index, eating habits (having/not having regularly scheduled meals) (yes/no; timing of meals), exercise habits (having/not having regularly scheduled exercise more than 30 min [twice times or more per week] for 1 year or more) [16] (yes/no), hobbies and/or group-activities (yes/no; type of activity), total walking-time and total sedentary-time per day, presence of chronic disease currently under treatment (yes/no), use of hypnotics (yes/no; types), and use of other medications (yes/no; types). Moreover, visual status (visual impairment/blindness; visual acuity; extent of visual loss), LP (with/without), cause of visual impairment (congenital/acquired; causative disease), age at onset of visual impairment, and morbidity length of visual impairment were investigated.

To estimate the participants’ sleep habits, they were asked about their bed- and wake-time, sleep duration, self-reported sleep onset, and variables related to waking after sleep onset (frequency of wake episodes and total number of wake periods during the night). In order to identify the presence of CRSWD and insomnia during the previous 3 months, items of diagnostic criteria other than those examined by actigram or sleep log were asked via telephone interviews by referring to the diagnostic criteria of the International Classification of Sleep Disorders – Third Edition (ICSD-3) (Table 1) [17].

**Table 1:** Diagnostic criteria of circadian rhythm sleep-wake disorders and insomnia

**Criteria of circadian rhythm sleep-wake disorders**		**Measurement scale**
1)	Progressive and day-by-day delay in bedtime/wake up time.(non-24-hour sleep-wake rhythm type)	yes/no; the range of daily delay
2)	Irregular sleep episodes at least three times during a 24-hour period.(irregular sleep-wake rhythm type)	yes/no; the duration of each sleep episodes and a total amount of sleep during a 24-hour period
3)	Presence of a stable advance (earlier timing) in bedtime/wake-up time relative to the general population and a difficulty staying awake until the desired bedtime or a difficulty remaining asleep until the desired time.(advanced sleep- wake phase type)	yes/no
4)	Presence of a stable delay (later time) in bedtime/wake up time relative to the timing required to maintain the schedule of social life and a difficulty falling asleep at the desired time or a difficulty waking up at the desired time.(delayed sleep-wake phase type)	yes/no
**Criteria of insomnia**		**Measurement scale**
5)	Difficulty falling asleep at night.	yes/no; less than once per week/ once or twice per week/ three or more per week^a^
6)	Difficulty maintaining sleep at night.	
7)	Waking up earlier than desired.	
8)	Difficulty staying awake and/or dozing off during the day.	
9)	Feeling of fatigue and/or difficulty in concentrating during day.	yes/no
10)	Presence of symptoms for at least three months.	yes/no

^a^These items correspond to the criteria 5-8

Based on the diagnostic criteria of the ICSD-3, CRSWD and insomnia were defined as follows: participants who answered “yes” to questions 1 or 2 and “three or more times per week” to questions 5, 6, or 7 and 8, 9, or 10 were classified as having a non-24-h/irregular sleep-wake rhythm type. We unified these two disorders because frequent bouts of sleep during the day caused by a non-24-h sleep-wake rhythm [18, 19] and those caused by irregular sleep-wake rhythm type could not be differentiated without use of an actigram or sleep log. Participants who answered “yes” to question 3 and “three or more times per week” to questions 7 and 8, 9 or 10 were classified as having advanced sleep-wake phase type, while those who answered “yes” to question 4 and “three or more times per week” to questions 5 and 8, 9 or 10 were classified as having delayed sleep-wake phase type. In this study, the presence of non-24-h/irregular sleep-wake rhythm type, advanced sleep-wake phase type, or delayed sleep-wake phase type was defined as “possible CRSWD”. Participants who answered “yes” and “three or more times per week” to questions 5, 6, or 7, and 8, 9, or 10 were classified as having insomnia.

### Statistical analysis

Before the main analysis, descriptive variables between participants and non-participants were compared using data obtained from a 2013 survey, and analyzed with the Mann–Whitney U test and chi-square test. After estimating the prevalence of CRSWD and insomnia, factors associated with these two disorders were examined using a series of logistic regression analyses. Age, gender, visual status, LP, social status, residential status, presence of treated diseases, exercise habits, and use of hypnotics were used as independent variables. All variables were initially examined in univariate models. In order to control for confounding factors and to determine the main correlates, multivariate logistic regression analyses were subsequently conducted for all variables showing *p* < 0.10 in the univariate models. Statistical tests for estimating odds ratios and confidence intervals (CI) were based on the likelihood ratio statistics. In these logistic regression models, the visually impaired individuals with possible CRSWD (*n* = 52) and those with insomnia symptoms (*n* = 45) were treated as targeted groups, and the visually impaired individuals who had neither CRSWD nor insomnia symptoms (*n* = 60) were treated as a reference group. Statistical analyses were conducted using the Statistical Package for the Social Sciences (SPSS) version 24.0 J (IBM SPSS, Inc., Tokyo, Japan).

## Results

### Comparison of descriptive characteristics between participants and non-participants

Participants (*n* = 157) were older (participants 59.5 ± 17.0 years vs. non-participants 54.4 ± 18.4 years, z = 3.26, *p* = 0.001) and were older age at the onset of visual impairment compared to non-participants (19.4 ± 20.8 years vs. 14.2 ± 18.8 years, respectively; z = 2.82, *p* = 0.005) (*n* = 343). No significant differences were found between participants and non-participants in terms of the other descriptive variables, including factors relating to visual impairment (Table 2).

**Table 2:** Comparison of descriptive characteristics between participants and non-participants

		Participants (*n* = 157)	Non-participants (*n* = 343)	Statistics	
Age (yrs)		59.5 ± 17.0	54.4 ± 18.4	Z = 3.26	*p* = 0.001
Gender	Female	61 (31.0)	136 (69.0)	χ (1) = 0.03	*p* = 0.866
	Male	96 (31.7)	207 (68.3)		
Social status	Employed/student	105 (30.2)	243 (69.8)	χ (1) = 0.77	*p* = 0.382
	Unemployed	52 (34.2)	100 (65.8)		
Body Mass Index (kg/m^2^)		22.9 ± 4.3	22.9 ± 3.8	Z = 0.65	*p* = 0.517
Visual status	Visual impairment	59 (31.1)	131 (68.9)	χ (1) = 0.00	*p* = 0.980
	Blindness	98 (31.6)	212 (68.4)		
Light perception	With	80 (32.3)	168 (67.7)	χ (1) = 0.17	*p* = 0.682
	Without	77 (30.6)	175 (69.4)		
Cause of visual impairment	Congenital	84 (33.5)	167 (66.5)	χ (1) = 0.10	*p* = 0.318
	Acquired	73 (29.3)	176 (70.7)		
Age at the onset of visual impairment		19.4 ± 20.8	14.2 ± 18.8	Z = 2.82	*p* = 0.005
Morbidity length of visual impairment		40.2 ± 21.7	40.3 ± 21.8	Z = 0.08	*p* = 0.934
Living alone		36 (33.0)	75 (67.0)	χ (1) = 0.18	*p* = 0.672
Living with family member		121 (30.9)	268 (69.1)		
Exercise habits	Yes	65 (28.6)	162 (71.4)	χ (1) = 2.85	*p* = 0.091
	No	92 (33.7)	181 (66.3)		

Date are expressed as mean ± standard deviation or *n* (%)

### Descriptive characteristics among visually impaired individuals

Descriptive characteristics of the study population are presented in Table 3. The study population consisted of 61 females (38.9%) and 96 males (61.1%) aged 61.0 ± 16.4 (mean ± SD) years. Of the visually impaired individuals, 115/157 (73.2%) were employed and/or students, 77/157 (49.0%) had no LP, and 34/157 (21.7%) used hypnotics. Specifically, 73.9% of visually impaired individuals with CRSWD (17/23) and 64.7% of those with insomnia (11/17) reported using hypnotics.

**Table 3:** Descriptive characteristics of the participants (*n* = 157)

		All visually-impaired individuals
Age (yrs)		61.0 ± 16.4 (range: 14-88)
Gender	Female/Male	61 (38.9) / 96 (61.1)
Social status	Employed or student/ Unemployed	115 (73.2) / 42 (26.8)
Body Mass Index (kg/m^2^)		22.8 ± 3.3 (range:15.2-34.8)
Visual status	VI/BL	59 (37.6) / 98 (62.4)
Light perception	With/Without	80 (51.0) / 77 (49.0)
Cause of visual impairment	Congenital/Acquired	84 (53.5) / 73 (46.5)
Age at the onset of visual impairment		19.1 ± 20.7 (range:0-75)
Morbidity length of visual impairment		41.9 ± 21.4 (range:6-79)
Living alone		37 (23.6)
Living with family member		120 (76.4)
Public assistance	With/without	113 (72.0)
Exercise habits	Yes/No	93 (59.2) / 64 (40.2)
Use of hypnotics	Yes/No	34 (21.7) / 123 (78.3)
Bedtime (h:m)		22:43 ± 01:16 (range: 19:00-27:00)
Wake-up time (h:m)		06:02 ± 01:04 (range: 03:00-09:00)
Sleep duration (hrs:m)		05:53 ± 01:17 (range: 03:00-11:00)
Sleep onset latency (min)		31.3 ± 39.6 (range: 1-195)
Wake-up after sleep onset (min)		35.8 ± 50.0 (range: 3-360)

Date are expressed as mean ± standard deviation or *n* (%)

*VI* visual impairment, *BL* blindness

### Prevalence of CRSWD and insomnia

The prevalence of CRSWD in the visually impaired individuals was 33.1% (95% CI: 25.8–40.5%) (*n* = 52). CRSWD included individuals with non-24-h/irregular sleep-wake rhythm type (*n* = 42, 26.8%; 95% CI: 19.8–33.7%), advanced sleep-wake phase type (*n* = 6, 3.8%; 95% CI: 0.8–6.8%), and delayed sleep-wake phase type (*n* = 4, 2.5%; 95% CI: 0.1–5.0%). The prevalence of insomnia in the study population was 28.7% (*n* = 45, 95% CI: 21.6–35.7%). The prevalence of CRSWD in the visually-impaired individuals with and without LP were as follows: non-24-h/irregular sleep-wake rhythm type, 15.0% vs. 39.0%; advanced sleep-wake phase type, 1.3% vs. 6.5%; and delayed sleep-wake phase type, 1.3% vs. 3.9%. Moreover, the prevalence of insomnia was 23.4% in the visually impaired individuals without LP and 33.8% in those with LP. In the present study, there were no cases of comorbidities with possible CRSWD and insomnia.

### Factors associated with CRSWD among visually impaired individuals

In the visually impaired individuals, univariate analyses revealed that the following five items were significantly associated with CRSWD: Blindness (*p* = 0.001), absence of LP (*p* < 0.001), living alone (*p* = 0.026), presence of diseases being currently treated (*p* = 0.001), and use of hypnotics (*p* = 0.002). In addition, three factors showed a marginally significant association with CRSWD: Older age (*p* = 0.059), unemployment (*p* = 0.075), and no exercise habits (*p* = 0.092). Multivariate logistic regression analysis using variables showing *p* < 0.10 in the univariate model revealed that the absence of LP, unemployment, living alone, and use of hypnotics were significantly associated with CRSWD (Table 4).

**Table 4:** Logistic regression analyses on associated risk for the existence both of circadian rhythm sleep disorder and insomnia

			Possible circadian rhythm sleep-wake disorders (n = 52)							Possible insomnia (n = 45)					
			Crude			Adjusted				Crude			Adjusted		
		*n=112*^a^	OR	95% CI	*P*	OR	95% CI	*P*	*n=105*^b^	OR	95% CI	*P*	OR	95% CI	*P*
Age			1.02	0.99 - 1.05	0.06					1.00	0.98 - 1.03	0.82			
Gender	Female	22/47	1.00						15/39	1.00					
	Male	30/66	0.89	0.45 - 2.01	0.95				30/66	1.33	0.60 - 2.99	0.49			
Visual status	VI	9/38	1.00						23/50	1.00					
	BL	43/75	**4.33**	**1.80 - 10.41**	**<0.01**				22/55	0.78	0.36 - 1.70	0.54			
Light perception	with	14/54	1.00			1.00			27/66	1.00					
	without	38/59	**5.17**	**2.30 - 11.61**	**<0.01**	**8.61**	**3.01 - 24.66**	**<0.01**	18/39	1.24	0.56 - 2.75	0.60			
Social status	Employed/student	35/85	1.00			1.00			32/80	1.00					
	Unemployed	17/28	2.21	0.92 - 5.29	0.08	**5.68**	**1.72 - 18.78**	**<0.01**	13/25	1.63	0.66 - 4.01	0.29			
Living alone	No	36/89	1.00			1.00			33/84	1.00					
	Yes	16/24	**2.94**	**1.14 - 7.60**	**0.03**	**3.57**	**1.17 - 10.88**	**0.03**	12/21	2.06	0.78 - 5.43	0.14			
Disease currently treated	No	13/47	1.00						20/52	1.00					
	Yes	39/66	**3.78**	**1.69 - 8.46**	**<0.01**				25/53	1.43	0.66 - 3.11	0.37			
Exercise habits	Yes	25/64	1.00						31/68	1.00					
	No	27/49	1.92	0.90 - 4.07	0.09				14/37	0.73	0.32 - 1.65	0.44			
Use of hypnotics	No	35/91	1.00			1.00			34/88	1.00			1.00		
	Yes	17/22	**5.44**	**1.84 - 16.07**	**<0.01**	**7.33**	**2.00 - 26.90**	**<0.01**	11/17	2.91	0.99 - 8.60	0.05	2.91	0.99 - 8.60	0.05

*OR* odds ratio, *CI* confidence interval, *VI* visual impairment, *BL* blindness

^a^The numerator indicates the number of visually impaired individuals with possible CRSWD (*n* = 52), and the denominator indicates the total number of individuals with possible CRSWD and those who had neither CRSWD nor insomnia symptoms (*n* = 60). ^b^The numerator represents the number of visually impaired individuals with possible insomnia (*n* = 45), and the denominator presents the total number of individuals with insomnia and those who had neither CRSWD nor insomnia symptoms (*n* = 60)

In the logistic regression models, those without both possible CRSWD and possible insomnia (*n* = 60) were classified as controls

### Factors associated with insomnia among visually impaired individuals

Among the visually impaired individuals, univariate analyses revealed that only one item, use of hypnotics, was marginally associated with insomnia. (*p* = 0.053). Multivariate logistic regression analysis also revealed that the use of hypnotics was marginally associated with insomnia (*p* = 0.053); however, no factors were found to be significantly associated with insomnia (Table 4).

## Discussion

This is the first nationwide epidemiological survey on the prevalence of CRSWD and insomnia, and their associated factors, specifically targeting visually impaired Japanese individuals via detailed telephone interviews.

The result showed that the prevalence of CRSWD in visually impaired Japanese individuals was slightly lower than that previously reported in a study conducted in a Western country (41.7%) [9], but apparently higher than that in the general Japanese population aged 15–54 years (0.13%) [20]. In addition, the prevalence of the non-24-h/irregular sleep-wake rhythm type (26.8%) was slightly higher than that reported by Leger et al. [2] (19%, consisting of 18% non-24-h sleep-wake rhythm type and 1% irregular sleep-wake rhythm type); this discrepancy could be ascribed to the difference in diagnostic procedures between studies. In the present study, we unified these two disorders because we were unable to determine whether frequent daytime sleep episodes were due to a non-24-h sleep-wake rhythm or to an irregular sleep-wake rhythm [18, 21]. Furthermore, in the current study population, the prevalence of the advanced sleep-wake phase type was slightly higher than that of the delayed sleep-wake phase type, but lower than that found by Lockley and colleague (13.4–22.0%) [6, 9, 10]; these differences across studies could be attributable to methodological differences in the assessment of circadian rhythm sleep-wake disorders. In the present study, we classified advanced and delayed sleep-wake phase disorders based on sleep characteristics without assessment of an endogenous circadian phase marker; therefore, the prevalence of these disorders may have been misidentified.

The non-24-h/irregular sleep-wake rhythm type was highly prevalent in the visually impaired individuals without LP; the percentage in this population was equivalent to that reported by Flynn-Evans et al. (39.0%) [9] and clearly higher than that in those with LP. Thus, an absence of light perception was inferred to be one of the risk factors of the non-24-h/irregular sleep-wake rhythm type. However, it is possible that participants who were currently classified as having LP by self-reporting could not perceive light sufficiently to maintain the circadian cycle [15]. Thus, further study should examine the extent to which light perception affects the circadian cycle in visually impaired individuals with LP.

The prevalence of insomnia among visually impaired Japanese individuals was lower than that reported in a previous study [2], in which insomnia was evaluated according to the minimum criteria in the 1st edition of the ICSD and was found to be in the range of 39–47% [2]. Although the reason for this discrepancy is unclear, it may be due to differences in the nosology and/or diagnostic criteria of insomnia between the ICSD-3 and the 1st edition of the ICSD [22]. Specifically, the diagnostic criteria for the ICSD-3 include the frequency (at least three times per week) and duration (at least 3 month) of insomnia and its associated daytime symptoms being different from the minimum criteria in the 1st edition of the ICSD, which requires only insomnia and its associated daytime symptoms. The rigorous criteria of ICSD-3rd might have contributed to the lower prevalence of insomnia in this study.

The most striking result of our study was that the factors differed between CRSWD and insomnia. In the multivariate analysis, the absence of LP, unemployment, living alone, and use of hypnotics were extracted as factors associated with CRSWD, whereas the use of hypnotics was only marginally associated with insomnia in the current study population. Among the factors associated with CRSWD, the absence of LP was most strongly associated with the disorder. Defective light perception due to photosensitive retinal ganglion cell dysfunction can result in loss of photic input to the suprachiasmatic nuclei, which clearly contributes to the formation of external desynchronization, leading to CRSWD [19, 23]. Thus, our result corroborates the notion that the absence of LP is an important risk factor for CRSWD in visually impaired individuals without LP [4, 6, 9, 19]. In addition, a lack of employment, possibly relating to decreased habitual daily activities, might have contributed to desynchronization of the participants’ circadian rhythms, as has been shown previously [4, 10]. Conversely, the non-24 cycling could not enable individuals to gain regular employment. In the visually impaired individuals living alone, it is also possible that a lack of communication with family members might contribute to dysregulated circadian rhythms [12–14]. The causal relationship between CRSWD and the use of hypnotics could not be identified in the present cross-sectional survey. However, the presence of nocturnal sleep disturbance due to CRSWD may have motivated the visually impaired individuals to use hypnotics.

Our results also demonstrated that no factors associated with insomnia in the visually impaired individuals, other than use of hypnotics, were extracted. In the general population, the presence of insomnia has been reported to be associated with female gender, older age, living alone, and the presence of comorbid medical conditions [24]. A possible reason for the discrepancy between the visually impaired individuals and the general population is the psychological and physiological characteristics specific to visual impairments; namely, that visually impaired individuals might have psychological/physiological problems [25, 26] that are more highly associated with hypnotic use than their demographic characteristics.

Several limitations of this study should be noted. First, although we surveyed the population by referring to the diagnostic criteria of the ICSD-3 to identify participants who had “possible CRSWD,” neither an endogenous circadian marker nor an actigraphy were used to diagnose CRSWD in this study. Hence, the non-24-h sleep-wake rhythm and irregular sleep-wake rhythm could not be differentiated, and the prevalence of these disorders may have been misestimated. Previous studies measured the secretion of urinary 6-sulfatoxymelatonin and cortisol, which accurately reflect circadian rhythm disruption [9, 10]. In future studies, these measurements should be performed in order to obtain a more accurate diagnosis of CRSWD. Second, we were unable to identify the morbidity length of CRSWD and insomnia because most visually impaired individuals could not recall the onset of CRSWD or insomnia. Third, the accuracy of information on LP is questionable because self-reporting was used to assess the degree and nature of the impairment. Consequently, detailed information on the nature of visual impairments that determine ocular or cortical blindness was unobtainable. Although the results of some previous studies have shown that self-reported data related to visual disability show at least moderate agreement with data from objective assessments [27, 28], objective data (i.e., ophthalmologic measurements such as electroretinographic testing) are preferred to examine the relationship between LP and CRSWD. In addition, the questions used in this telephone interview were obtained from the ICSD-3 and based on the diagnostic criteria of CRSWD and chronic insomnia, but their validity was not been confirmed; thus, the outcomes should be interpreted with caution. Fourth, the relatively small sample size could have limited the identification of factors associated with CRSWD and insomnia. Initially, because of the high prevalence of these disorders, we expected to extract important associated factors. An important caveat is also the lack of a control group to compare the results found in the visually impaired individuals. Thus, future studies should examine the differences among the three groups by including a control group and stratifying visually impaired individuals into two groups—those with and without LP. Additionally, the low response rate may have been a possible source of bias. In contrast, low response rates with telephone interviews are increasingly becoming a problem in recent years, likely because of the multitude of such surveys being conducted [29]. In most of these surveys, the potential respondents accept or reject participation before being asked specific questions. Thus, it is unlikely that the respondents had any personal interests involved, and this reduces any possible selection bias. More recently, factors related to willingness to participate in telephone surveys have also been identified [30]. In the future, suitable procedures should be considered to motivate the target population to be more actively engaged in this kind of studies based on the Japanese context.

Furthermore, in the present study, the possibility of response bias might be limited because there were significant but small differences in demographic characteristics (i.e., no significant differences were found in the descriptive variables, except for age at investigation and at onset of visual impairment) between participants and non-participants. Fifth, information was lacking on the use of melatonin/melatonin agonists in the study participants, which may have affected the status of CRSWD. Finally, it should be noted that approximately 5 years have passed since the study was conducted, and the information is not necessarily up to date.

## Conclusions

CRSWD and insomnia were observed in 33.1 and 28.7% of visually impaired Japanese individuals, respectively. The results of this study raised a possibility that increasing physical activity, social contact and getting a regular job is helpful for preventing CRSWD and insomnia in this population.

## Supplementary Information

**Additional file 1.** Interview Form. Interview form developed to conduct this survey.

## Data Availability

The data for the present study will not be shared publicly as participants were informed at the time of providing consent that only researchers involved in the project would have access to the information they provided.

## Abbreviations

CRSWD: Circadian rhythm sleep-wake disorders
LP: Light perception
ICSD-3: International Classification of Sleep Disorders – Third Edition
CI: Confidence intervals
JFB: Japan Federation of the Blind
