# Supplementary material for: Prevalence and associated factors of circadian rhythm sleep-wake disorders and insomnia among visually impaired Japanese individuals
Source: BMC Public Health. 2021 Jan 6;21:31. doi: 10.1186/s12889-020-09993-8 (PMC7789312; doi:10.1186/s12889-020-09993-8)
Supplement: Supplementary file 1 — Additional file 1. Interview Form. Interview form developed to conduct this survey. [file 12889_2020_9993_MOESM1_ESM.pdf]

Additional File 1: Interview Form

Reception No：	Receprion date：
Name：	Birthday： yrs
Tel：	☐Male ☐Female
Address：	Height： Weight:

☐ Do you work regularly or periodically?	☐ Yes→(1)	☐ No
(1) Do you work from home or other place?	☐ Home	☐ Away from home
(2) Does your work involve desk job or physical job?	☐ Office	☐ Physical
Job details:		
(3) How many days do you work per week?	___________days	per week：
	Start of a workday：	
	Finish time：	

☐This interview will take about 25 minutes. Is this convenient for you?

Yes No （Convenient date and time： ）

◇First ask about the eye health condition. You can choose wether to participate or not. If there are items you do not want to answer, please say so.

1. Provide the grade of your disability certificate:		
☐ Grade 1 (Visual acuity of <0.02)		
☐ Grade 2		
☐ I do not have a certificate. ☐ Others（ ）		
2. Provide your present visual acuity (If the acuity of each eye is provided, please write each.)		
☐ Visual acuity:		
☐ Counting fingers	☐ Hand motion	
☐ Total blindness → Is the visual acuity of 0 correct?	☐ Yes	If Yes → 5.
	☐ No (the visual acuity:)	
3. Do you perceive light?	☐ Yes	
	☐ No	If No → 5.
4. How does the visual field look like?		
☐ No problem at all	☐ The edge is not visible.	
☐ The center is not visible.	☐ The field is not homogeneous.	

5. What is the cause of visual impairment?

a. Congenital b. Acquired

1. Traumatic injury 2. Glaucoma 3. Retinal detachment 4.Diabetic retinopathy 5. Macular degeneration

6. Hemorrhage (☐Retina ☐Brain) 7. Cataract 8. Anoxia at birth（Retinopathy of prematurity）

9. Retina (Blastoma) 10. Ocular albinism･Oculocutaneous albinism･Albino 11. Retinitis pigmentosa

12. Aneurysm 13. Brain tumor 14. Behcet's disease 15. Leber disease 16.Optic atrophy

17. Other ( )

6. When did you get the certificate? At the age of (）or（ years ago）

7. When did the present condition occur? At the age of (）or（ years ago）

8. Have you been taking any medication for the eye? Yes No

（Medication name）

9. Do you currently have any disease that affects your lifestyle? Yes No

（Disease name）

10. Have you recently taken any sleep medication? Yes No

（Medication name）

11. Have you been taking any medication other than the above-mentioned medication? Yes No

（Medication name）

12. Meals

Do you eat your meals regularly?	No	Yes			
Do you eat breakfast everyday?	No	Yes	If Yes →	hours	minutes
Do you eat lunch everyday?	No	Yes	If Yes →	hours	minutes
Do you eat dinner everyday?	No	Yes	If Yes →	hours	minutes

13. Do you use public support? Yes No

How many times a week do you use the support? times per week

How many hours a week do you use the support in total? hours per week

14. Free time and club activities

Do you involve yourself in cultural activities such as karaoke, singing in chorus, writing tanka poems, igo, and shogi? Yes No

Details

How many times a week do you participate in the cultural activities? times per week

15. Exercise habits

Do you currently exercise regularly? Yes No

If Yes → a. Walking activities

b. Yoga, stretch training, or resistance training

c. Radio gymnastics, runnning, swimming, social dance, sound teble tennis, or volleyball

d. Other ( )

The type of exercise:

How many days a week do you do the exercise? days per week

How long do you exercise? hours minutes

How long have you been exercising? years months

16. Walking activities in daily life

How many days a week do you continuously walk for over 10 minutes? days per week

How much walking do you do in a day? Specify the amount of time spent walking as well as the average number of steps taken (working, traveling, and shopping)

During work: hours minutes

Traveling: hours minutes

Average number of steps taken a day: steps

17. Sitting activities in daily life

How long do you sit and lie down in a day (except when sleeping)? Specify the amount of time spent sitting during work and freetime.

During work: hours minutes

During traveling: hours minutes

During free time: hours minutes

◇The sleep habits and sleep-related symptoms during the last one month		
What time do you usually go to bed during the last one month?	AM･PM ＿:＿.	
What time do you usually wake up during the last one month?	AM･PM ＿:＿.	
How long do you usually sleep during the last one month?	Approximately＿ hours minutes a day on average	
How long does it take for you to sleep?	Approximately＿ minutes	
Do you have difficulty trying to sleep at night?	Yes	No
YES→Frequency?<br>(0. None 1. Less than once a week 2. Once to twice a week 3. Three times a week or more)	0 1 2 3	
Do you sometimes wake up in the middle of the night?	Yes	No
YES→Frequency?<br>(0. None 1. Less than once a week 2. Once to twice a week 3. Three times a week or more)	0 1 2 3	
If YES→･How long do you stay awake for?	minutes	
Do you sometimes wake up too early in the morning then have difficulty going back to sleep?	Yes	No
YES→Frequency?<br>(0. None 1. Less than once a week 2. Once to twice a week 3. Three times a week or more)	0 1 2 3	
When waking up in the morning, do you sometimes still feel tired?	Yes	No
YES→Frequency?<br>(0. None 1. Less than once a week 2. Once to twice a week 3. Three times a week or more)	0 1 2 3	
Do you feel very sleepy during the day?	Yes	No
YES→Frequency?<br>(0. None 1. Less than once a week 2. Once to twice a week 3. Three times a week or more)	0 1 2 3	
Do you feel sluggish, tired, or suffer from poor concentration during the day?	Yes	No
Do you snore?	Yes	No
Do you sometimes have difficulty breathing while sleeping at night?	Yes	No
Have you felt like not breathing while sleeping or has someone pointed it out?	Yes	No
Do your legs sometimes twitch or feel itchy while sleeping at night?	Yes	No

◇Timing･time window to go to sleep and wake up

Is bedtime and wake-up time almost fixed? “Almost fixed” should be considered as around the same time ocurring within one hour.

(1) Bedtime	Fixed	Not fixed
(2) Wake-up time	Fixed	Not fixed
(3) Do you have any problem with bedtime or wake-up time? For example, you are late for school, work, or an appointment, do you feel tired when you had trouble sleeping at night?	Yes	No

If (1) the bedtime and/or (2) the wake-up time is not fixed, go to the question items of free-running type/irregular sleep-wake type.

If (1) and (2) are fixed, but the bedtime is somewhat late (after 2 am) or the wake-up time is somewhat late (after 8 am), go to delayed sleep-wake phase type.

If (1)and (2) are fixed, but the bedtime is somewhat early (before 10 pm) or the wake-up time is somewhat early (before 5 am), go to advanced sleep-wake phase type.

Free-running type

Does the window period of bedtime and wake-up time get increasingly delayed every day?	Yes	No
By how many hours does your sleep and wake-up time get delayed every day?	______ hours	
Does your sleep and wake up pattern get delayed and happen to be late until daytime or nighttime?	Yes	No
Which time of the day do you feel less sleepy? Morning (10 am –12 pm), afternoon (1–4 pm), and evening (4–8 pm)?	Morning Afternoon Evening	
Since when did you observe this pattern?	Since years old or years ago	
Are there any triggers (causes) that come to mind?	Yes No	

Irregular sleep-wake type

Are your sleep times not limited to the nighttime and do you sleep during the day (i.e . taking naps) as well?	Yes	No
How many hours is your longest sleep in a day?	______ hours	
How many hours is your shortest sleep in a day?	______ hours	
Combining the amount of sleep taken during the night and day, how many hours is your total sleep time?	______ hours	
Which time of the day do you feel less sleepy? Morning (10 am –12 pm), afternoon (1–4 pm), and evening (4–8 pm)?	Morning Afternoon Evening	
Since when did you observe this pattern?	Since years old or years ago	
Are there any triggers (causes) that come to mind?	Yes No	

Delayed sleep-wake phase type

At what time do you prefer to go to sleep and wake up?	Going to sleep at	
	Waking up at	
Can you wake up at the desired time?	No, I can't wake up.	Yes, I can wake up.
Can you go to sleep at the desired time?	No, I can't go to sleep.	Yes, I can go to sleep.
Can you fix your sleeping and waking up pattern at the desired time you prefer?	No, I can't.	Yes, I can.
Which time of the day do you feel less sleepy? Morning (10 am –12 pm), afternoon (1–4 pm), and evening (4–8 pm)?	Morning Afternoon Evening	
Since when did you observe this pattern?	Since years old or years ago	
Are there any triggers (causes) that come to mind?	Yes	No

Advanced sleep-wake phase type

At what time do you prefer to go to sleep and wake up?	Going to sleep at	
	Waking up at	
Can you wake up at the desired time?	No, I can't wake up.	Yes, I can wake up.
Can you go to sleep at the desired time?	No, I can't go to sleep.	Yes, I can go to sleep.
Can you fix your sleeping and waking up pattern at the desired time you prefer?	No, I can't.	Yes, I can.
Which time of the day do you feel less sleepy? Morning (10 am –12 pm), afternoon (1–4 pm), and evening (4–8 pm)?	Morning Afternoon Evening	
Since when did you observe this pattern?	Since years old or years ago	
Are there any triggers (causes) that come to mind?	Yes	No
